# The Cost of Caring: Burnout, Compassion Satisfaction and Quality of Life Among Nurses in Chronic Care Settings in Ghana

**DOI:** 10.1002/nop2.70923

**Published:** 2026-10-09

**Authors:** Collins Atta Poku, Jeremiah Kofi Asante, Felix Kyei‐Fram, Pius Oppong, Abigail Araba Bafoa Acquah, Sarah Akuyo Brown, Doris Hagan

**Affiliations:** ^1^ Department of Nursing, School of Nursing and Midwifery KNUST Kumasi Ghana; ^2^ Nursing and Midwifery Training College Cape Coast Ghana; ^3^ Department of Public Health Nursing, School of Nursing and Midwifery KNUST Kumasi Ghana

**Keywords:** burnout, chronic care units, compassion satisfaction, emotional exhaustion, nursing workforce, quality of life

## Abstract

**Aim:**

To examine the effects of compassion satisfaction and burnout on the quality of life (QoL) of nurses working in chronic care settings.

**Design:**

A cross‐sectional design.

**Methods:**

A simple random sampling approach was used to select 360 respondents (269 included in the analysis) from a targeted population of 650. Data were collected between February 2025 and June 2025 using validated tools to assess nurses' QoL, compassion satisfaction and burnout in chronic care units. Pearson correlation and Hayes' PROCESS Macro Mediation analyses were conducted at a *p*‐value < 0.05.

**Results:**

The findings showed that respondents generally reported high compassion satisfaction (*n* = 42.43), moderate burnout and moderate quality of life (*n* = 2.73). Compassion satisfaction positively influenced QoL (*β* = 0.1402, *p* < 0.001), while emotional exhaustion negatively affected it (*β* = −0.0452, *p* < 0.0001). Burnout partially mediated the relationship between compassion satisfaction and QoL (*F*_(4, 264)_ = 20.01, *p* < 0.001). The interaction term between compassion and age was also significant (*β* = −0.0207, *p* < 0.0479), indicating that age moderated the effects of compassion satisfaction on QoL.

**Conclusion:**

Compassion satisfaction influenced the nurse's burnout and quality of life. The findings emphasise the importance of fostering a supportive work environment to allay burnout and enhance quality of life.

**Implications for the Nursing Profession/or Patient Care:**

Interventions aimed at increasing compassion satisfaction could effectively reduce burnout and improve nurses' overall QoL. Nurse managers should also consider compassion‐focused leadership and opportunities for meaningful patient engagement as part of a holistic approach to staff well‐being.

**Impact:**

The study's findings highlight the need to design mentorship programmes that foster compassion satisfaction to improve quality of life and, indirectly, cushion burnout. Early‐career nurses could build resilience during formative professional years, thereby improving compassion satisfaction and quality of life.

**Reporting Method:**

STROBE checklist‐guided reporting.

**Patient or Public Contribution:**

No patient or public contribution.

## 1 Background

Providing safe, high‐quality care is a fundamental goal of healthcare systems worldwide, and the nursing workforce plays a vital role in achieving it. However, they face a critically demanding work environment, especially in chronic care settings. Nurses in chronic wards integrate technical and non‐technical skills in daily interactions with patients and relatives to foster a supportive healing environment through empathy (Laari 2024; Prineas et al. 2021). This role often overwhelms them and may affect nurses' emotional, psychological and physical judgement, impacting their quality of life (QoL) (Todaro‐Franceschi 2024).

Compassion satisfaction, the positive emotional state derived from helping others, has been identified as an important factor contributing to nurses' well‐being. The pleasure of helping others recover from illness or trauma can improve their QoL (Todaro‐Franceschi 2024; Yu et al. 2021; Zhang et al. 2018). Nurses gain professional satisfaction in this way, which improves their QoL and is reflected in their engagement in activities that improve working conditions and, in turn, patient care. In addition, nurses often cope with the stressful nature of their work by adjusting their working conditions personally, managing both on‐ and off‐duty work and seeking professional counselling (Bahari et al. 2022). Despite gaining professional satisfaction from their duties and providing healthcare, nurses are continuously exposed to events following patients' deaths, posing a significant risk of burnout (Kelly 2020).

In contrast, burnout, characterised by emotional exhaustion, has been recognised as a pervasive issue in healthcare, with nurses particularly vulnerable (Geuens et al. 2021). Al‐Worafi (2024) describes burnout as resulting from extensive exposure to demanding work triggers, such as role ambiguity, workload, understaffing and inadequate peer support. Burnout is often viewed as a consequence of a disconnection between the high standards of nursing performance and the level of support nurses receive from their employers. It has been identified as resulting from the cumulative effects of numerous stressors, leading to a loss of coping ability; hence, addressing this aspect is essential in nursing practice to improve patient care and nurses' QoL (Hennes 2017).

Recent studies suggest that high levels of compassion satisfaction can buffer against burnout, fostering resilience and QoL among healthcare professionals (Buonomo et al. 2022; Ruiz‐Fernández et al. 2020, 2021). However, while researchers have studied the individual effects of compassion satisfaction and burnout on nurses' QoL, less is known about burnout's mediating role in the relationship between compassion satisfaction and QoL, especially in challenging work environments marked by high patient‐to‐nurse ratios and demanding tasks associated with chronic illness care.

In Ghana, nurses in tertiary hospitals face demanding work conditions, including high patient workloads, complex care for chronic illnesses and emotional stress from prolonged patient interactions (Nyande et al. 2025; Gmayinaam et al. 2024). These challenges may increase burnout risk and negatively affect nurses' QoL and the quality of care. Given that the nursing profession is increasingly recognised for its emotional demands, it is essential to understand how burnout mediates this relationship, as this may offer insights to improve nurses' well‐being and the care they provide. Stakeholders in the healthcare industry tasked with providing appropriate resources to improve nurses' working conditions can rely on this study's findings to enhance the practice environment and improve the quality and safety of healthcare delivery.

### 1.1 Aim

The study examined the effects of compassion satisfaction and burnout on nurses' QoL in chronic care settings.

### 1.2 Theoretical Framework

The study was anchored in the Compassion Fatigue‐Satisfaction Model (Figley 1995; Stamm 2012), which provides a framework for understanding how caregiving roles simultaneously generate fulfilment and strain. The model posits that professionals who sustain empathetic relationships with clients experience two outcomes: compassion satisfaction (the positive reinforcement and meaning derived from helping) and compassion fatigue, which encompasses burnout and secondary traumatic stress. These constructs jointly influence the caregiving workforce's QoL.

In the context of chronic care units, nurses operate under conditions characterised by prolonged patient contact, high emotional exhaustion, limited resources and systemic stressors. These conditions align closely with the model's emphasis on the dialectic of reward and depletion inherent in helping professions. Nurses may derive deep satisfaction from improving the lives of patients with long‐term conditions, yet also face exhaustion from relentless care demands.

This study framed compassion satisfaction as a protective factor that can mitigate burnout's negative impact on nurses' QoL. It also positioned burnout not only as an individual psychological state but also as a systemic outcome shaped by workload, role ambiguity and organisational culture. The model thereby allowed for an integrated analysis of how positive and negative occupational experiences interact to determine nurses' overall well‐being.

## 2 Methodology

### 2.1 Design

A cross‐sectional design was used to examine the role of burnout in the relationship between compassion satisfaction and QoL among nurses in the chronic care settings of a Teaching Hospital between February 2025 and June 2025.

### 2.2 Study Area

The study was conducted in the chronic care settings of a Teaching Hospital. The hospital was chosen for its high volume of essential cases and prolonged patient admissions, which expose nurses to high‐stress conditions that lead to burnout.

### 2.3 Population

The target population included nurses in the chronic care settings of a Teaching Hospital. It comprised about 650 nurses working in the chronic care settings. Nurses who had worked in the unit for at least a year were included in the study. The study excluded nurses who were not directly involved in patient care and those who declined to participate.

### 2.4 Sampling Method and Sample Size

The study used simple random sampling. This sampling strategy ensured that readily available nurses participated in the survey. G*Power (v3.1) for a priori sample size estimation for the regression step, testing the incremental variance explained by the mediator (linear multiple regression: fixed model, *R* ^2^ increase), was used to estimate the sample size (Erdfelder et al. 1996). Based on prior work and aiming to detect a small‐to‐moderate effect, an effect size of *f* ^2^ = 0.04 was specified. G*Power indicated a required sample size of *N* = 360 nurses, ensuring representativeness and statistical power. However, only 269 nurses completed and returned the questionnaires.

### 2.5 Measures

The study used three self‐administered, validated questionnaires: the Maslach Burnout Inventory (MBI), the Professional Quality of Life Scale (ProQoL) Version 5 and the World Health Organisation Quality of Life (WHOQOL)—BREF.

#### 2.5.1 Maslach Burnout Inventory

Burnout was assessed using an adapted version of the MBI (Human Services Survey) developed by Maslach and Jackson (Maslach et al. 1997). This survey tool comprises three dimensions with 21 items on a 7‐point Likert scale, ranging from ‘never’ to ‘every day’, with ratings ranging from 0 to 6. To assess burnout, the MBI measured three dimensions: emotional exhaustion, depersonalisation and personal accomplishment. For emotional exhaustion, a respondent's total score of 17 or less indicates low‐level burnout, 18–29 inclusive indicates moderate burnout, and 30 or more indicates high‐level burnout. Previous studies have reported Cronbach's alpha values of 0.7 or higher (Forné and Yuguero 2022; Lin et al. 2022).

#### 2.5.2 Professional Quality of Life Scale Version‐5 (ProQoL)

Nurses' compassion satisfaction was measured using the ProQoL‐5 (Hemsworth et al. 2018). This validated scale comprises 30 items on a 5‐point Likert scale, ranging from 1 *(never)* to 5 *(very often)*, measuring compassion satisfaction (10 items), burnout (10 items) and secondary traumatic stress (10 items). ProQoL‐5 measures compassion satisfaction among nurses in clinical practice. Only the compassion satisfaction subscale was used for the study. The respondents' total score is obtained by adding the item scores. The overall test score is 50. A score of 22 or less indicates a low level of compassion satisfaction; 23–41, an average level; and 42 or more, a high level. Previous studies reported Cronbach's alphas ranging from 0.75 to 0.88 (Inocian et al. 2025; Shdaifat et al. 2023).

#### 2.5.3 World Health Organisation Quality of Life (WHOQOL)—BREF

The QoL of nurses was assessed using a single‐item measure of self‐rated overall QoL from the WHOQOL‐BREF (The WHOQOL 1998), which assesses respondents' overall perception of their QoL. This tool uses a 5‐point Likert scale from 1 (very poor) to 5 (very good), with higher scores indicating better perceived QoL. The tool was analysed as a single‐item measure of nurses' QoL. The tool has been used in previous studies and has reported a reliability score of > 0.7 (Maqsood et al. 2021; Orszulak et al. 2022).

The Cronbach alphas of the scales after the pre‐test were as follows: MBI, *α* = 0.78; ProQoL Scale, *α* = 0.74; and WHOQOL–BREF, *α* = 0.82.

### 2.6 Data Collection

The researchers administered the questionnaires during routine hospital visits. The nurse manager contacted the respondents after sampling. The researchers explained the study and data collection procedure to respondents to obtain consent. Respondents who signed the written consent received the questionnaires and were instructed and encouraged to complete and return them in a sealed envelope to ensure anonymity. This ensured inclusivity and minimised non‐response.

### 2.7 Data Analysis

The data collected from the respondents were analysed using SPSS version 27. Data were screened for completeness, and questionnaires with missing responses were excluded from the analysis. The socio‐demographic characteristics of respondents were presented using descriptive statistics (frequencies, percentages, means and standard deviations). Bivariate analysis (Pearson product–moment correlation) examined relationships among compassion satisfaction, burnout dimensions and QoL among nurses working in chronic wards. A moderated mediation analysis was conducted using Hayes' PROCESS macro (Model 5). The model examined whether burnout mediated the association between compassion satisfaction and QoL, and whether age moderated this pathway. Covariates were selected a priori based on theoretical consideration and evidence from previous studies. Continuous variables were mean‐centred before analysis. Indirect effects were tested using bootstrapping with 5000 resamples and bias‐corrected 95% confidence intervals. Regression coefficients are presented as unstandardised estimates.

### 2.8 Ethical Considerations

In accordance with the Declaration of Helsinki, the Committee on Human Research Publication and Ethics (CHRPE) of Kwame Nkrumah University of Science and Technology granted ethical approval (CHRPE/AP/0133/2). The hospital's management granted permission to conduct the study before the survey. Respondents signed consent forms after the researchers explained the entire process. Respondents' data were also treated with the utmost confidentiality. The researchers ensured voluntary participation, anonymity and confidentiality through an anonymous survey that collected no identifying information.

## 3 Results

### 3.1 Socio‐Demographic and Work Characteristics of Nurses

The study yielded a response rate of 74.7% (*n* = 269) among the anticipated 360 nurses invited to participate. The data analysis was therefore based on the 269 completed responses. Table 1 details the socio‐demographic and work characteristics of nurses. The largest age group was 20–35 years, comprising 46.5% (*n* = 125) of the sample. Most respondents were female (*n* = 205, 74.3%) and had up to 5 years of experience (*n* = 152, 56.5%). Most of the respondents (*n* = 206, 76.6%) worked 40 h or more per week.

**TABLE 1:** Descriptive statistics for socio‐demographic and work characteristics.

Variable	Category	*N*	%
Age	20–35 years	125	46.5
	35–50 years	88	32.7
	Above 50 years	56	20.8
Gender	Male	64	25.7
	Female	205	74.3
Years at work	Up to 5 years	152	56.5
	6–10 years	82	30.5
	Above 10 years	35	13.0
Hours per week	Less than 40 h	63	23.4
	40 h or more	206	76.6

### 3.2 Compassion Satisfaction, Burnout and QoL of Nurses

Table 2 details the compassion satisfaction, burnout and QoL among nurses. The composite mean for compassion satisfaction was 42.43 (SD = 6.53), indicating a high level of compassion satisfaction. The mean scores for emotional exhaustion (18.99), depersonalisation (11.35) and personal accomplishment (32.34) indicated moderate levels of burnout. The composite mean score for nurses' QoL was 2.73 (SD = 1.60), indicating a moderate QoL.

**TABLE 2:** Mean scores of compassion satisfaction, burnout and QoL of nurses.

Variables	Dimension	Range	Mean	SD
Compassion satisfaction		5–50	42.43	6.53
Burnout	Emotional exhaustion	0–54	18.99	7.65
	Depersonalisation	0–30	11.35	8.22
	Personal accomplishment	0–48	32.34	6.66
Overall QoL		1–5	2.73	1.60

### 3.3 Relationship Between Compassion Satisfaction, Burnout and QoL Among Nurses

Table 3 details the relationships among nurses' age, compassion satisfaction, emotional exhaustion, depersonalisation, personal accomplishment and QoL. There was a significant negative correlation between compassion satisfaction and emotional exhaustion (*r* = −0.258, *p* < 0.001), between compassion satisfaction and depersonalisation (*r* = −0.418, *p* < 0.001) and a significant positive correlation with personal accomplishment (*r* = 0.559, *p* < 0.001) and QoL (*r* = 0.418, *p* < 0.001). Emotional exhaustion also showed a significant negative correlation with QoL (*r* = −0.309, *p* < 0.001). Meanwhile, age showed no significant correlation with any variable under study.

**TABLE 3:** Correlations between compassion satisfaction, burnout and QoL.

	1	2	3	4	5	6
1. Age	—					
2. Compassion satisfaction	0.003	—				
3. Emotional exhaustion	−0.062	−0.258**	—			
4. Depersonalisation	−0.073	−0.418**	0.493**	—		
5. Personal accomplishment	0.022	0.559**	−0.094	−0.356**	—	
6. Quality of life	−0.042	0.418**	−0.309**	−0.221**	0.311**	—

** Correlation is significant at the 0.01 level (2‐tailed).

### 3.4 Moderated Mediation Analysis

Table 4 details the model, which showed that the relationship between emotional exhaustion (*Y*) and compassion satisfaction (*X*) was significant (*F*_(1, 267)_ = 19.01, *p* < 0.001), with an *R* ^2^ of 0.0665, indicating that compassion satisfaction accounted for approximately 6.65% of the variance in emotional exhaustion. The regression analysis showed that compassion satisfaction had a significant negative effect on emotional exhaustion (*β* = −0.3020, *p* < 0.001), indicating that higher levels of compassion satisfaction were associated with lower levels of emotional exhaustion.

**TABLE 4:** Mediation model for compassion satisfaction, burnout and QoL.

Path	*R* ^2^	Coefficient	SE	*T*	*p*	95% CI
CS → EE	0.0665	−0.3020	0.0693	−4.3602	0.0000	[−0.4383, −0.1656]
CS → QoL	0.2327	0.1402	0.0295	4.7554	0.0000	[0.0822, 0.1983]
EE → QoL		−0.0452	0.0117	−3.8746	0.0001	[−0.0682, −0.0222]
Age		0.7813	0.4382	1.7830	0.0757	[−0.0815, 1.6440]
Interaction (CS × Age) (Int_1)		−0.0207	0.0104	−1.9877	0.0479	[−0.0411, −0.0002]
**Conditional effects**						
Age (20–34) (Effects of *X* on *Y*)		0.1196	0.0208	5.7390	0.0000	[0.0785, 0.1606]
Age (35–50) (Effects of *X* on *Y*)		0.0989	0.0146	6.7508	0.0000	[0.0701, 0.1277]
Age (> 50) (Effect of *X* on Y)		0.0576	0.0206	2.7964	0.0055	[0.0170, 0.0981]
**Indirect effect**						
CS → EE → QoL		0.0137	0.0049		0.0001	[0.0052, 0.0242]

*Note:* *N* = 269; the paths were controlled for age; the displayed effects are standardised; the indirect effect is significant (*) when the 95% CI does not include 0.

Abbreviations: CS, compassion satisfaction; EE, emotional exhaustion; QoL, quality of life; *R* ^2^, coefficient of determination; SE, bootstrap regression standard error.

The second model assessed QoL as the outcome variable (*Y*), which was influenced by compassion satisfaction (*X*), emotional exhaustion (*M*) and age (*W*). The model fit was significant (*F*_(4, 264)_ = 20.01, *p* < 0.001), with an *R* ^2^ of 0.2327, suggesting that compassion satisfaction explained 23.27% of the variance in QoL. Compassion satisfaction positively influenced QoL (*β* = 0.1402, *p* < 0.001), while emotional exhaustion negatively affected it (*β* = −0.0452, *p* < 0.0001). Age, although positively related to QoL (*β* = 0.7813, *p* < 0.0757), was not statistically significant at the 0.05 level. The interaction term between compassion satisfaction and age was also significant (*β* = −0.0207, *p* < 0.0479), indicating that age moderated the effects of compassion satisfaction on QoL.

The conditional effects of compassion satisfaction on QoL were examined at three age percentiles (1st, 50th and 84th). At younger ages, compassion satisfaction had a stronger positive effect on QoL (Effect = 0.1196, *p* < 0.001 for age 20–35). In contrast, the effect decreased with age but remained significant (Effect = 0.0576, *p* = 0.0055 for age above 50). This suggests that the relationship between compassion satisfaction and QoL is stronger for younger nurses. The moderated mediation analysis indicated that emotional exhaustion partially mediated the relationship between compassion satisfaction and QoL, with a significant indirect effect, suggesting that higher compassion satisfaction reduces emotional exhaustion, which in turn positively affects QoL.

## 4 Discussion

The study assessed the effect of burnout on compassion satisfaction and QoL among nurses working in the chronic wards of a tertiary facility. The study posits that a relatively large proportion of the nursing workforce is younger. This matters for nursing management, as younger nurses may face unique challenges, including a higher risk of burnout due to inexperience or ineffective coping strategies, particularly in high‐stress clinical environments (Labrague and McEnroe‐Petitte 2018). The fact that 76.6% of respondents worked 40 h or more per week emphasises the significant workload that nurses face. This prolonged work duration, coupled with high demands, as reported by Li et al. (2021), can contribute to burnout and decreased QoL.

The moderate to high level of compassion satisfaction indicates that, while nurses feel optimistic about their ability to care for patients, they also experience high burnout, suggesting challenges in maintaining satisfaction. This phenomenon is particularly concerning in the nursing profession because of its well‐documented links to turnover (McDermid et al. 2020), absenteeism (Dyrbye et al. 2019) and decreased patient care quality (Eltaybani et al. 2021; Jun et al. 2021). Nurses' moderate QoL suggests that, while most feel relatively satisfied, a notable proportion may experience low life satisfaction, possibly due to professional stress or work–life balance challenges (Zhang et al. 2024).

Higher compassion satisfaction is associated with lower burnout. This finding supports previous studies indicating that increased job satisfaction can buffer against burnout (Wu et al. 2021). Further, compassion satisfaction was positively associated with QoL, supporting studies that found a direct link between job satisfaction and overall life satisfaction (Bahari et al. 2022; Saliya et al. 2024). However, the negative association between burnout and QoL further emphasises the importance of managing emotional exhaustion to improve health professionals' QoL. These findings also align with Ruiz‐Fernández et al. (2021), who reported that burnout significantly detracts from a nurse's well‐being, highlighting the need for interventions to reduce burnout and improve work–life satisfaction.

Age as a moderator in the relationship between compassion satisfaction and QoL showed that the positive association between the two is stronger among younger nurses and decreases with age. This finding aligns with previous studies (Kim and Ko 2018; Onyedibe et al. 2025) that found younger workers tend to exhibit higher levels of enthusiasm and job satisfaction. This age‐related moderation may reflect factors such as physical and emotional resilience and career experience, which typically increase with age. Older nurses may be accustomed to managing job demands, which could mitigate the impact of compassion satisfaction on their QoL (Alruwaili et al. 2024).

The mediation analysis, which showed burnout partially mediates the relationship between compassion satisfaction and QoL, is similar to Galiana et al.'s (2022) findings, which suggested that emotional exhaustion is a critical pathway through which compassion satisfaction influences well‐being. This finding highlights the importance of fostering environments that support compassion satisfaction, as it not only improves resilience but also enhances overall QoL.

## 5 Limitations

Limitations have been acknowledged. The cross‐sectional design limits the ability to infer causal relationships among the variables. Again, self‐reported measures may lead to social desirability bias. Moreover, the study was conducted in a single tertiary hospital, though many units in the Internal Medicine Department may affect the generalisability of the findings to other healthcare settings. Future research should explore how work environment factors and personal coping mechanisms further explain the pathways through which compassion satisfaction influences nurse well‐being. Also, exploring nurses' views on compassion satisfaction through a qualitative approach in chronic healthcare settings will shed further light on the concept.

## 6 Conclusions

Compassion satisfaction influenced nurses' burnout and QoL. Burnout partially mediated the relationship between compassion satisfaction and QoL. These findings emphasise the importance of fostering a supportive work environment to allay burnout and enhance QoL. Overall, organisational commitment is essential to supporting nurses in both their professional and personal lives, helping develop a sustainable healthcare workforce and ultimately improving patient care outcomes.

## 7 Implications for the Nursing Profession/or Patient Care

These findings have substantial implications for nursing and policy. Interventions aimed at increasing compassion satisfaction could effectively reduce burnout and improve nurses' overall QoL. Such interventions could include promoting supportive work environments, offering regular recognition and rewards for compassionate care, and providing professional development opportunities. Moreover, addressing burnout through stress management and counselling services would likely improve QoL and reduce the harmful effects, such as emotional exhaustion and depersonalisation. Meanwhile, because many nurses work long hours, offering flexible schedules or shift rotations might help reduce the negative association between long hours and nurses' well‐being. The moderating role of age suggests that interventions may need to be tailored to nurses' age groups, with younger nurses likely to benefit more from compassion‐focused interventions. Again, nurse managers should consider compassion‐focused leadership, recognition programmes and opportunities for meaningful patient engagement as part of a holistic approach to staff retention and well‐being. Promoting compassion satisfaction is not merely a personal resource but a managerial priority with measurable outcomes in nurse performance and health system efficiency.

## Author Contributions

**Collins Atta Poku:** writing – original draft, methodology, writing – review and editing, formal analysis, conceptualization, supervision. **Jeremiah Kofi Asante:** conceptualization, investigation, writing – original draft, methodology, writing – review and editing, data curation. **Felix Kyei‐Fram:** conceptualization, investigation, methodology, data curation. **Pius Oppong:** conceptualization, investigation, methodology, data curation. **Abigail Araba Bafoa Acquah:** conceptualization, investigation, methodology, data curation. **Sarah Akuyo Brown:** writing – original draft, methodology, supervision. **Doris Hagan:** supervision, formal analysis, writing – original draft.

## Funding

The authors have nothing to report.

## Ethics Statement

In accordance with the Declaration of Helsinki, ethical clearance was sought from the Committee on Human Research Publication and Ethics (CHRPE) of Kwame Nkrumah University of Science and Technology, Kumasi, Ghana (CHRPE/AP/0133/25), dated 24/02/2025. At the same time, permission to conduct the study was received from the Human Resource Unit of the KATH before the survey was conducted. Respondents in the study were required to sign consent forms after the entire process was explained to them. Respondents' data were also treated with the utmost confidentiality. The researchers ensured voluntary participation, anonymity and confidentiality through an anonymous survey that collected no identifying information.

## Conflicts of Interest

The authors declare no conflicts of interest.

## Data Availability

The data that support the findings of this study are available from the corresponding author upon reasonable request.
